# Dr. Ware on General Theurapeutics

**Published:** 1862-03

**Authors:** 


Dr. Ware on General Therapeutics.—The remarks that have been made on certain states of the skin naturally bring us to the subject of animal heat, the variations of which are chiefly connected with the skin and its sensations, whilst the influence of cold on these variations and consequently on the production, phenomena and course of disease, is constantly to be taken into view in its treatment. Although it is universally conceded that exposure in various ways to external temperature is one of the most efficient causes of disturbance of the health, we know very little of the laws according to which this takes place. In common opinion nothing is regarded as more certain than that disease is constantly produced, and that the symptoms of disease are constantly modified, by what is familiarly spoken of as "taking cold." Yet almost no exact knowledge is possessed of the mode in which this effect is brought about, or of the conditions of the body itself, or of the external agents on which it is supposed to depend. The common notions so confidently entertained concerning the whole matter afford us one of the most remarkable of the examples, only too common, in which we mistake the habitual use of certain forms of expression, which have become familiar, but to which we attach no definite idea, for a clear comprehension of the subject to which they relate.

The easy solution thus resorted to for the purpose of explaining the effects of "taking cold," is used on occasions of the most opposite kinds, and for very different diseases. It is employed without at all understanding the manner in which the animal heat is modified by the circumstances of the exposure, or of the manner in which the modification operates to the production of disease. The most common idea is that we take cold by the withdrawal, in some way, of the heat of the body. Yet it is manifest that this withdrawal takes place in such a different manner, in such a different degree, and under such different conditions, that there can be no uniformity in its *modus operandi* as a cause. Whilst, on the one hand, the same disease is produced by very different modes of exposure, on the other, the same mode of exposure will in other cases produce very different forms of disease. Thus, in one case where cold is taken, there has been but a momentary exposure of a small part of the body to a cause which can produce but a very slight loss of animal heat, as a draught of cold air; in another, a large quantity has been abstracted from the whole surface, as by remaining a long time in the water, or exposed to a high cold wind; in another case, no assignable impression of the kind has taken place at all, and yet, in each, disease has been produced. Colds, of apparently the same character, prevail under circumstances the most diverse, as in cold weather and warm weather, in dry weather and in damp, in uniform weather and in variable, in one kind of season and in its opposite.

The most reliable results we can extract from the vague mass of observation on this subject are, that sudden exposure, especially when the body has been much heated, the skin is in an excited state, and the body is insufficiently, or less than habitually clothed, or when the weather has suddenly changed from warm to cold, or from cold to warm, is a condition under which the cases we attribute to cold are very likely to arise; that the gradual, and still more the rapid abstraction of large quantities of animal heat, however this is brought about, are also likely to produce disease, but in this case of a more deep-seated, continuous and obstinate character, though not always more violent; but that the most extensive, though less obvious injurious effects of cold are found among those who are exposed through long and inclement seasons, with deficient protection by habitations, clothing and fuel, especially among the young. Is it to be observed, however, that there is no uniformity in the degree or kind of the effects which are thus produced, and that it is but a limited proportion of the persons exposed to these causes who are unfavorably affected by them. Hence we are obliged to infer that we can justly consider the disturbance of the animal heat as constituting but one of the conditions upon which depends the origin of the diseases usually attributed to taking cold.

We are not likely, then, to derive any considerable aid in the direction of treatment from our knowledge of the connection of the scientific history of animal heat with the production of disease, and we are obliged to depend mainly on the teachings of observation and experiment. In the early stages of acute diseases there is usually some over-production or accumulation of animal heat; this, when excessive, causes no little suffering and restlessness; and many of the other troublesome symptoms of the febrile condition seem closely associated with it, such as headache, pains in the back and limbs, want of sleep, and thirst. Whether the accumulation of heat is the cause of these other symptoms or not, it is very certain that the alleviation of the heat of the skin by cool air, cool sponging, the application of cold cloths or ice to the parts most affected, and cool drinks, not only give relief to the sensation of heat, but tend also to mitigate the other febrile symptoms. How much this relief actually contributes to the removal of disease, we can not say, but the quieting of pain and thirst and the production of sleep are always important objects to secure.

But throughout some acute diseases, and in the advanced stages of all, there is a diminished production of animal heat. Notwithstanding this, there may still remain a tendency to occasional exacerbations, but more limited in extent and continuance. In such cases the loss of heat becomes exhaustive, and should be guarded against by attention to food, clothing and the other hygienic conditions which will prevent this loss. The warmth of the body, as a whole, is to be watched and maintained. Yet there is in this state of things no inconsistency in the application of means for the relief of the temporary attacks of heat, locally or even generally. The main principle to be held in view is to prevent that general depression of the powers of the system which is so certainly the result of a permanent draught upon its efforts for keeping up the temperature of the body. The effects of this exhaustion are not always perceived by the sensation of cold, but often by a disturbance of some of the internal functions. Thus, a convalescent will experience a loss of appetite, headache, neuralgic pains, watchfulness, troublesome sleep, as well as other symptoms, from a considerable fall of temperature in his chamber, of which he is hardly sensible, but which a careful observation of the cause of the phenomenon will satisfy the physician has been due to cold—not to taking cold in the ordinary sense of the word, but to the general depression of vital force due to its disproportioned expenditure in the maintenance of temperature. This especially manifests itself in organs which have been the subjects of the disease, and hence the great relief often experienced from the application of direct warmth, and also of extra clothing, to such parts.

Perhaps the most common mistake in attending to this point, is to overlook the necessity of the access of fresh air to sick rooms, and to endeavor to maintain their warmth mainly by keeping out cold air, instead of warming that which is introduced. Breathing cool, or even cold air, is seldom injurious, and carries off but little heat, where it is pure and fresh, if the body be surrounded by a sufficient amount of non-conductors to preserve that which is generated.

It is sometimes observed that the external surface of the body is cool, while the patient experiences the sensation of heat. This is often noticed in common cases in a slight degree, but in some violent diseases it is exhibited in a very striking degree. The skin of the patient is cold, even icy cold, but he seems to himself to be burning with intense heat. No external means will warm the body, except as it will warm a corpse; it even seems more difficult than this—and it becomes cold again, as soon as applications are suspended. There may be an occasional and limited flush of heat, but for the most, the coldness remains till death, when the case is fatal; but then, the surface, which could not be warmed from without, during life, becomes gradually warm from within; and, in some cases certainly, the internal viscera are found very warm, and even at a higher than the normal temperature, if the body be opened. In this singular state of things, most noticeable in cholera, it would seem as if the animal heat was prevented from diffusing itself during life by some modification of the law of conduction dependent upon the presence of vitality, but on the cessation of life is permitted to obey the ordinary conducting powers of the textures.

Every one acquainted with cholera has probably observed, in the collapse of this disease, how useless was the attempt, so persistently made, to make the patients warm by hot-air baths, hot sand, hot bricks, etc., and how much discomfort was occasioned by the attempt; whilst, on the other hand, how grateful to them was cold sponging, the administration and application of ice, and the free admission of cold air to the skin and lungs, and this although the breath was sometimes itself cool. May it not be that the relief thus experienced has some connection with that modification of the conducting powers of the textures just suggested as a probable explanation of those singular phenomena; and some indirect connection also with the familiar fact that persons or parts which have been chilled or frozen are more speedily and safely relieved by cold applications than by warm. All such facts probably have a significant relation to the various phenomena of disease connected with cold and heat, and these sensations of the patient; and also with chilliness, shivering and ague turns, all of which are connected with these sensations, and with various disturbances of the nervous system.

In chronic diseases, the management in reference to animal heat enters more universally into the plan of treatment than in acute. In acute it is rather incidental; in chronic it is fundamental. A due equilibrium between the production and transmission of heat is essential to the perfect performance of all those functions on which, as formerly stated, the processes of recovery depend. The regulation of all the habits of life as to clothing, habitations, artificial warmth, climate, exposure, air and exercise, have some connection with the maintenance of this equilibrium, and although in a state of health, and to a certain degree under disease, we can endure a certain amount of departure from it, yet, in proportion as disease is present, the capacity for this endurance is diminished, and it becomes important to prevent the unfavorable effects of it. In a majority of chronic diseases, the heat-making and cold-resisting capacity appears to be impaired, and since, partly as the consequence, exhaustion, debility, and a defect in the power of recovery are the prevailing tendencies of these diseases, the regulation of all the habits which relate to this point should be a constant subject of regard.

Under the ordinary exposure of mankind, even in the hottest climates, the current of heat is from within outward, and it rarely happens that we do not part with more heat than we receive. In some seasons and in some countries the quantity lost is small, and of course there must be a large check upon those vital operations by which heat is developed. This check, however, seems to be, taken alone, seldom injurious, and the diseases of hot climates, so far as we can refer them to the influence of accumulated heat, are few as compared with those of cold. It is notorious that hot climates and hot seasons are healthy, except so far we can trace the origin of disease to causes generated by the action of heat upon substances outside of the human body. But it is not so with the influence of cold, for although many other causes co-operate with and intensify its agency, we yet know that by itself alone it deteriorates health and produces disease.

In managing most chronic diseases, therefore, it is the demand upon the body for its heat, and the necessity of arranging the habits of the patient with a view to this, that we have to consider. Not only the ordinary demand for the evolution of heat continues, but generally a diminished capacity exists for this evolution, and consequently a liability to be injured and exhausted by it, which does not exist in health. Doubtless there is a great difference in the power of different individuals in this respect, but there are few in whom the capacity for resisting cold is not diminished by all chronic disease. In practice, it is always safest to err on the side of too much precaution than too little, especially in the matter of clothing. Persons will often suffer in their sensations from too much, but rarely in their diseases.

This is true even of the general clothing of individuals in temperate climates in health; and I believe it to be a fact, and an important one, that the most common fault in such climates is to endeavor to get along with too little clothing, and that it is really a safer rule to wear as much clothing as we can bear without discomfort, than as little as we can without it. Doubtless there are individuals with regard to whom this is not true, and cases in which they are exhausted and debilitated by much clothing, whilst they are refreshed and invigorated by diminishing it. But this I am convinced, after long attention to the point, is rather exceptional, and yet frequent enough to make a due regard to it proper in every case. There are many general rules in practice, but no universal ones, and this is so here.

I can only now state this general principle of the sanative effects of preventing the dispersion of the animal heat. Its practical management runs out into so many details which require to be modified by constitution, circumstances of exposure, and character and stage of disease, that they must be left to the knowledge and good sense of each practitioner. The greatest error perhaps on this point arises from the disposition to substitute external warmth by artificial heat, and the exclusion of air and confinement within doors, for adequate clothing. The effect of this error is to deprive patients with chronic diseases, and even persons with simply feeble health, of the invigorating influence of external air. Bad and variable as our climate is, there are few individuals who may not, by the careful and gradual cultivation of the habit, acquire the power of going abroad in all seasons and almost all weathers, not only without danger but with positive benefit. More persons induce imperfect health by the habits of seclusion engendered by the fear of taking cold, than are actually injured by taking cold. No doubt an artificial state of the system in this particular is produced in many persons, and no sudden change of habit would be safe. Their management should be cautious and delicate, but there are probably few in whom the change might not be brought about. This is even true of a class of complaints—those of the throat and lungs—usually regarded as peculiarly liable to injury from exposure. The use of the respirator is especially valuable in such cases, and is almost universally sufficient to prevent injury. Even the irritating and harrassing influence of the east winds of spring is thus signally mitigated. This instrument is also capable of performing an important office in husbanding the animal heat, and many persons are thus kept warm by it and secured from the influence of cold, by a much less amount of protection than would otherwise suffice.

The application of artificial heat to particular organs, and also the prevention of the escape of the natural heat from them, is often found of important aid in relieving many of the sensations of disease, and probably also in promoting their recovery. A diseased organ, as well as a diseased system, may be unfavorably affected by the exhaustion of its heat, and derive a consequent benefit by preventing it. I have already referred to the good effects of guarding organs which have suffered from acute disease, or exposure during convalescence. The examples of a corresponding advantage to organs affected with chronic disease are even more numerous. Local pains, both rheumatic, neuralgic and from chronic inflammation, affections of the kidneys, bowels and uterus, and many of the throat and lungs, furnish numerous examples of this sort.

It is obvious how many other particulars of treatment are suggested by these considerations, and yet how impracticable it is to enter into them in a consideration whose object is to illustrate merely the principles upon which treatment is to be conducted, and not to enter into a full account of its details. —*Boston Medical and Surgical Journal.*

